# Baculovirus Infection Triggers a Shift from Amino Acid Starvation-Induced Autophagy to Apoptosis

**DOI:** 10.1371/journal.pone.0037457

**Published:** 2012-05-21

**Authors:** Wei Wei, Zhongchao Gai, Hui Ai, Wenxian Wu, Yongbo Yang, Jianxin Peng, Huazhu Hong, Yi Li, Kaiyu Liu

**Affiliations:** College of Life Science, Central China Normal University, Wuhan, People's Republic of China; George Mason University, United States of America

## Abstract

Autophagy plays a central role in regulating important cellular functions such as cell survival during starvation and control of infectious pathogens. On the other hand, many pathogens have evolved mechanisms of inhibition of autophagy such as blockage of the formation of autophagosomes or the fusion of autophagosomes with lysosomes. Baculoviruses are important insect pathogens for pest control, and autophagy activity increases significantly during insect metamorphosis. However, it is not clear whether baculovirus infection has effects on the increased autophagy. In the present study, we investigated the effects of the *Autographa californica* nucleopolyhedrovirus (AcMNPV) infection on autophagy in SL-HP cell line from *Spodoptera litura* induced under amino acid deprivation. The results revealed that AcMNPV infection did not inhibit autophagy but triggered apoptosis under starvation pressure. In the early stage of infection under starvation, mitochondrial dysfunction was detected, suggesting the organelles might be involved in cell apoptosis. The semi-quantitative PCR assay revealed that the expression of both *p35* and *ie-1* genes of AcMNPV had no significant difference between the starved and unstarved SL-HP cells. The western blot analysis showed that no cleavage of endogenous Atg6 occurred during the process of apoptosis in SL-HP cells. These data demonstrated that some permissive insect cells may defend baculovirus infection via apoptosis under starvation and apoptosis is independent of the cleavage of Atg6 in SL-HP cells.

## Introduction

Autophagy is an essential cellular process that mediates continuous recycling of intracellular components such as organelles and protein aggregates and becomes an alternative source of energy when nutrients are scarce. Novel roles of autophagy in embryogenesis, development, cellular defense and cell death as well as immune response of animals have emerged from recent studies [1]–[4]. Its newfound ability to consume disease-inducing invaders has even spawned a new term – xenophagy [5]. Virus and other pathogens can be engulfed into a double membrane vesicle called autophagosome and then are digested in lysosomes fused with autophagosome to form single-membrane autolysosomes [2]. However, viral subversion and inhibition of host cell autophagy have been also documented for several viruses [6], [7]. Some viruses have evolved mechanisms inhibiting the formation of autophagosomes or the fusion of autophagosomes with lysosomes to protect their survival and replication [8]. Under normal conditions, the baseline of autophagy is low in insect cells. However, amino acid deprivation or rapamycin promoted autophagy activity in insect both *in vivo* and *in vitro* [9]–[10]. It is not clear whether baculovirus infection enhances autophagy in insect cells, especially under starvation pressure.

In the previous study, we observed that some non-permissive insect cell lines underwent apoptosis when challenged with baculovirus [11]. Numerous evidences indicated that cross-talking between autophagy and apoptosis is common in mammalian and insect cells [12], [13]. Therefore, we are also very interested in exploring whether this shift from autophagy to apoptosis occurs in permissive insect cells infected with baculovirus under starvation pressure.

Autophagy-related gene, *Beclin-1/Atg6*, expresses constitutively in mammalian cell lines and tissues, and the cleavage of Atg6 protein was reported to be involved in apoptosis in mammalian cells [14]. The expression of exogenous *Atg6* gene has not been reported in lepidoptera insect cells, and whether the Atg6 protein undergoes cleavage during apoptosis in Lepidopteran cell remains unclear.

In the present study, we demonstrated that autophagy was induced in SL-HP cell line derived from *Spodoptera litura* via amino acid deprivation, which was permissive to baculovirus infection [15], the influences of baculovirus infection on the cross-talking between autophagy and apoptosis were investigated, and the possible mechanism of the shift from autophagy to apoptosis was discussed.

## Materials and Methods

### Ethics Statement

The experimental materials contained two mice for the preparation of antibody. The research protocol was approved by the Institutional Review Board at Central China Normal University in China (CCNUIRB) on April 21, 2011, and the number of permission approval was CCNUIRB2011008. The experiment was performed by Wei.

### Reagents and solutions

Grace's insect medium and fetal bovine serum (FBS) were from Gibco (Invitrogen-Gibco, Grand Island, NY, USA). Hoechst 33342 was purchased from Sigma–Aldrich. Substrate of caspase-3 (Ac-DEVD-AFC) was obtained from BD Biosciences. Lyso-Tracker Red and Mito-Tracker Red CMXRos were obtained from Molecular Probes (Eugene, OR). MitoPT™ JC-1 kit was purchased from ICT (Immunochemistry Technologies, LLC, Bloomington, MN). Insect balanced salt solution (IBSS, 75 mM NaCl, 5 mM CaCl_2_, 55 mM KCl, 2.6 mM MgCl_2_·6H_2_O, 2.8 mM MgSO_4_·7H_2_O, 4.2 mM NaHCO_3_, 7.3 mM NaH_2_PO_4_·H_2_O, 10 mM glucose, pH 6.4) was prepared and sterilized with a 0.22 µm filter in our labortary.

### Insect cell culture, induction of autophagy and baculovirus infection

SL-zsu-1 cell line, a gift from Zhongshan University in China, was established from the ovaries of *Spodoptera litura* [16]. This cell line was not susceptible but apoptotic to baculovirus infection. However, it became permissive to baculovirus infection after high passages during a few years, which was named as SL-HP cell line [15]. These two cell lines were cultured in Grace's medium with 10% FBS at 28°C.

*Autographa californica* multiple nucleopolyhedrovirus (AcMNPV), *Anagrapha falcifera* multiple nucleopolyhedrovirus (AfMNPV), *Spodoptera litura* multicapsid nucleopolyhedrovirus (SplitMNPV) were stored in our laboratory. The recombinant baculovirus Ac-PH-GFP-actin in which the *eGFP* gene was fused with *actin* from *Drosophila melanogaster* under the control of the AcMNPV polyhedrin promoter was constructed using Bac-to-Bac expression system (Invitrogen) in our laboratory.

To understand the effects of baculovirus infection on starvation-induced autophagy, SL-HP cells were cultured in complete medium with 10% FBS over night. Then the medium was replaced with IBBS after washing twice with IBBS and the cultures were divided into three groups. In the tested group 1(AcMNPV-infected SL-HP cells under starvation pressure), the medium was replaced with IBBS. After 1.5 h of the treatment (starvation), AcMNPV was added into the cultures at moi = 5. The virus was removed after 1 h of absorption and the cells were washed twice with IBSS. Then the infected cells were cultured in IBSS for different time periods. In the tested group 2 (Starved SL-HP cells), the cells were treated the same as the tested group 1 except that baculovirus was replaced with an equal volume of medium. In the control group (Unstarved SL-HP cells), the cells were grown in the complete medium with 10% FBS.

### Analysis of autophagy

In mammalian cells, punctual spots of Atg8 increase when autophagy activity has been enhanced in the early stages of starvation, but the abundance of Atg8 declines significantly with the extension of time [17]. Since we have cloned *Helicoverpa armigera* Atg8 gene (GenBank accession No: JQ739159, an orthology of LC3), which is a good molecular marker of autophagy, the punctual spots of the expressed GFP-HaAtg8 was used to monitor autophagy. The protocols were briefly described as following: the ORF of HaAtg8 gene was amplified via RT-PCR with primers HaAtg8-F and HaAtg8-R (Table 1) using cDNA extracted from *H. armigera* larvae as template. The PCR product was inserted into plasmid pEGFP-C1 after the cleavage of both PCR fragments and plasmid with enzymes *Hind* III and *BamH* I from New England Biolabs. Following this, the OpIE2 promoter was amplified from pIZ-V5/His (Invitrogen) and inserted into plasmid pEGFP-HaAtg8 cleaved by restriction enzymes *Bgl* II and *Sac* I. SL-HP cells were transfected via the plasmid using cell-fectin (Invitrogen) according to the manufacturer's protocol. Then the medium was replaced with insect balance salt buffer for the amio acid starvation at 24 h of post-transfection. In addition, baculovirus was added into the starved cells at M.O.I. of 5 and incubated for 1 hour after 1.5 h of starvation. Following this, the IBSS containing baculovirus was removed and IBSS was added after the cells had been washed twice with IBSS. The alteration of GFP-HaAtg8 distribution was observed under fluorescence microscope.

**Table 1:** PCR primers used in this study.

Primer	Sequence^a^	Final target
PIZ-Atg6F	gg GGTACC ATG AGT GAT TCA AAG ACG TTC GT	Expression in insect cells
PIZ-Atg6R	gc TCTAGA CCT TGT GGC TCA GAT TTG TCC TC	Expression in insect cells
PET-Atg6F	cgc GGATCC ATG AGT GAT TCA AAG ACG TTCGT	Expression in bacteria
PET-Atg6R	ccc AAGCTT TTG TGG CTC AGA TTT GTC CTC	Expression in bacteria
PIE2-Atg6F	ccc AAGCTT TCA TGA TGA TAA ACA ATG TAT GGT G	Location in insect cells
PIE2-Atg6R	cgc GGATCCCC TTG TGG CTC AGA TTT GTC CTC	Location in insect cells
AcMNPV IE1-F	CGA CGA CAA CGA CTA CAA	Semi-PCR at cDNA level
AcMNPV IE1-R	CTC ACA TAC GGC GAT ACA A	Semi-PCR at cDNA level
AcMNPV P35-F	TCC ACG ATA GCA TCA AGT	Semi-PCR at cDNA level
AcMNPV P35-R	GAG CAA ACG GCA CAA TAA	Semi-PCR at cDNA level
Actin-F	GCG CGG CTA CTC GTT CAC TAC C	Semi-PCR at cDNA level
Actin-R	GGA TGT CCA CGT CGC ACT TCA	Semi-PCR at cDNA level
HaAtg8-F	ccc AA GCT TCG GGC GGT GGA GGG ATG AAA TTC CAA TAT AAA GAA G	Cloning
HaAtg8-R	cg GGATCC TTA ATA TCC ATA TAC ATT CT	Cloning
Total	14	7

a : The lower-case letters indicate the corresponding protective bases of the restriction enzymes. The underlining letters indicate the cleavage sites of the restriction enzymes.

Amino acid starvation (1–3 h) was commonly used to induce autophagy as positive control as described [18]. Although monodansylcadaverine (MDC) staining of autophagic vesicles is not very specific if cells have been fixed, it is specific marker of autolysosomes and autophagosomes in living cells and can be used to reveal autophagic vesicles (autolysosomes and autophagosomes) [9], [18]. In our experiments, when the cells were induced for autophagy with IBSS (the test group 2) or IBSS plus baculovirus infection (the tested group 1) for different time periods, MDC (in dimethyl sulfoxide-ethanol) was added to the cultures at a final concentration of 50 µM for 15 min at 28°C. After washing twice with IBBS, the cells were viewed under a Nikon fluorescence microscopy 2000 to detect autophagic vesicles.

It is well known that the increase in quantity of lysosomes and lyso-enzyme activity is well correlated to autophagy activity. To evaluate changes in quantity of lysosomes between control groups and the tested groups, the cells were stained with Lyso-Tracker Red according to the instruction provided by the company and observed using fluorescence microscope. To analyze the potential ability of lysosomes to degrade cytoplasmic materials and organelles in SL-HP cells between the control and the tested groups, lysosomal activity was measured as follows: cells cultured in the control groups and the tested groups were harvested by centrifugation, and rinsed once with IBBS. A total of 1×10^6^ cells from each sample were lysed with 30 µl H_2_O containing 1% Triton X-100 for 30 min on ice. Then, the lysate was used for the analysis of acid phosphatase (ACP) by using disodium phenyl phosphate as the substrate according to the method as described previously [19]. The experiments were performed in triplicate.

To detect autophagic vesicles (autolysosomes and autophagosomes), the cells in the control groups and the tested groups were fixed with glutaraldehyde and osmium tetroxide, and dehydrated in ethanol, respectively. Cell pellets were embedded in epon resin and sectioned with an ultramicrotome at a 70 nm thickness. Samples were observed with a transmission electronic microscope (TEM) after staining with uranyl acetate+lead citrate for enhanced protein and lipid staining.

### Detection of apoptosis

The most important changes during cell apoptosis are cell shrinkage, chromatin condensation, formation of cytoplasmic blebs and apoptotic bodies [18], [19]. To observe alterations in cellular morphology, Hoechst staining was used for the assay of chromatin condensation and the formation of apoptotic bodies. Analysis of DNA fragmentation and caspase-3 activity was performed according to the previously reported method [19].

### Detection of mitochondrial membrane potential and the release of cytochrome c from mitochondria to cytoplasm

MitoPT™-JC1 (JC-1) reagent is used to detect the change of mitochondrial membrane potential. JC-1 staining was performed according to the instruction provided by the supplier. MitoPT™-JC1 was examined with excitation at 488 nm and emission at >590 nm.

To analyze the release of cytochrome c, the co-localization of mitochondria with cytochrome c was performed. In brief, the cells in the test group 1 and 2 were stained with Mito-Tracker Red CMXRos according to the method provided by company in order to localize mitochondria and fixed with 4% of paraformaldehyde. Immunofluorescence to localize cytochrome c was performed using a rabbit anti-horse cytochrome c monoclonal antibody as the first antibody and a FITC-labeled goat anti-rabbit as the second antibody diluted at 1: 1000, respectively. The co-localization of mitochondria with cytochrome c was observed under a confocal laser microscope.

### Analysis of the expression of *ie-1* and *p35* using semi-quantitative PCR

The *ie-1* and *p35* of AcMNPV are an apoptotic gene and an anti-apoptotic gene, respectively. AcMNPV IE-1 is a 67-kDa dimeric DNA-binding protein and has been implicated in triggering apoptosis during infection. IE-1 may contribute directly by transactivation of host prodeath genes, by induction of a host DNA damage response, or by alteration of the cell cycle [20]. Baculovirus p35, a broad-range inhibitor of the caspase family, blocks apoptosis induced by TNF, Fas, glucocorticoids, radiation, DNA-damaging agents and nerve growth factor withdrawal, and inhibits caspases-1, -2, -3, -4, -6, -7, -8 and –10 *in vitro* [21]. Both the genes are expressed in the immediately early stage of infection and the balance between their expressions regulates baculovirus replication and apoptosis [22]. Thus, the analysis of the differential expression of *ie-1* and *p35* was required in order to understand the mechanism of apoptosis induced in the permissive SL-HP cells under starvation pressures. The protocols were described as follow: At 6 h of post–infection with baculovirus under normal or starvation condition, the cells were harvested and the total RNA was prepared using RNeasy mini kit (QIAGEN). The contamination of DNA in the total RNA sample was digested with DNase I according to the method as previously described [23]. The RNAtag (gggtctagagctcgagT17) primer was used for first-strand cDNA synthesis. Each of cDNA samples was divided into three groups and used for the RT-PCR of *ie-1*, *p35, actin3A*, respectively. The expression of *ie-1* and *p35* was assessed by the analysis of semi-quantitative PCR for the test groups and control groups at 6 h of post-infection.

### Detection of the cleavage of *S. litura* Atg6 protein

Since *S. litura Atg6* (SlAtg6) sequence is not available in the GenBank, *Bombyx mori Atg6* (BmAtg6, putative) sequence (GenBank accession No: FJ416328.1 and HQ651091.1)was used as a referred template [24]. BmAtg6 was PCR-amplified from the cDNA of *B. mori* larvae using the PIZ-Atg6F and PIZ-Atg6R, or the PET-Atg6F and PET-Atg6R primers. Then the PCR products were cloned into the pIZ-His/V5 (Invitrogen) and pET-28 a (+) (EMD Biosciences) vectors in-frame with the tag of the vector to generate the pIZ-Atg6 and the pET-Atg6, respectively. The pET-Atg6 plasmid-expressed protein in *Escherichia coli* BL21 (DE3) strain formed inclusion bodies. Atg6 protein was purified by using Ni-NTA column (QIAGEN) after adding 8 M of urea to solubilize those inclusion bodies. Then anti-BmAtg6 mouse serum was produced by immunizing mouse with the purified protein cut from SDS-PAGE gels. The BmAtg6 with ie-2 promoter was PCR-amplified using the PIE2-Atg6F and PIE2-Atg6F as primers and the plasmid pIZ-Atg6 as a template. Then the PCR products were subcloned in-frame with eGFP into pEGFP-N1 (Clonetech). *Lymantria dispar* LD652 cells (High efficiency in the transfection) were transfected with the recombinant plasmid pIE-Atg6-GFP to study the localization of lepidopteran Atg6 protein. All the primer sequences in the study were listed in table 1.

Apoptosis induced in SL-HP cells with actinomycin D at the final concentration of 1 µg/ml was used as a positive control. SL-HP cells were harvested at 12 h in the presence or absence of actinomycin D and lysed with cell lysis buffer purchased from Beyond company (Shanghai, China). After the determination of protein concentration using BCA method, the samples were loaded into the wells of the 10% SDS-PAGE gel at an equal quantity. The proteins were transferred onto a nitrocellulose (NC) membrane after the electrophoresis. Then western blot was performed using the conventional method. Anti-BmAtg6 mouse serum and alkaline phosphatase-conjugated goat anti-mouse second antibody was diluted at 1: 1000 (Santa Cruz Technology, CA USA), respectively.

### Statistical analysis

Statistical analysis of differences among the groups was performed using student's t test. *p*<0.05 was considered statistically significant, and all data are presented as mean ± SE.

## Results

### All the four Baculoviruses replicated in SL-HP cells

**Figure 1:**
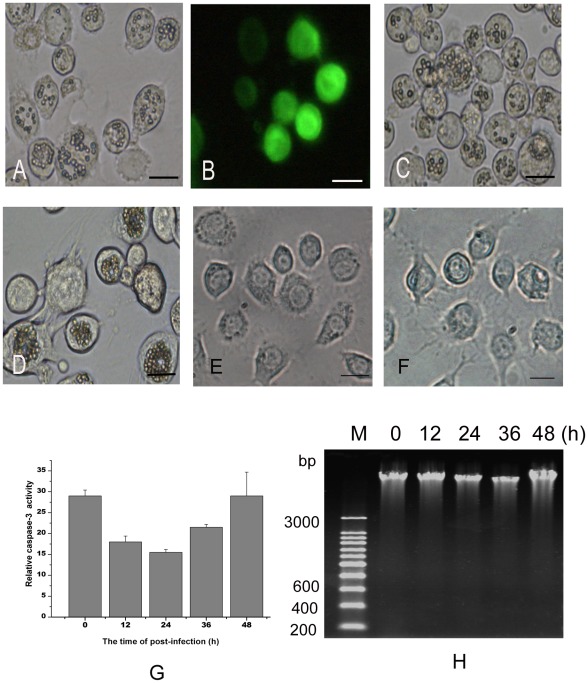
Replication of four baculoviruses in *S. litura* SL-HP cells cultured in complete medium with 10% FBS. (A) AcMNPV infection for 48 h, (B) Ac-PH-GFP-actin infection for 48 h, (C) AfMNPV infection for 48 h, (D) SplitMNPV infection for 72 h, (E)Unstarved cells, (F) Starvation for 24 h, (G) Relative caspase 3 activity in AcMNPV-infected SL-HP cells at different time points of post-infection, (H) DNA ladder assay for AcMNPV-infected SL-HP cells at different time points of post-infection (0, 12, 24, 36, 48 h). Bars = 20 µm.

The infections of AcMNPV, AfMNPV, SplitMNPV and the recombinant Ac-PH-GFP-actin were tested in SL-HP cells cultured in Grace's supplemented with 10% FBS. Our results revealed that all the four viruses could replicate in the SL-HP cells and many polyhedra or green fluorescent proteins were observed in the infected cells (Figure 1A, B, C and D). No apoptotic characteristics were detected in the infected SL-HP cells, such as the formation of apoptotic bodies, activation of procaspase-3 and fragmentation of DNA (Figure 1A, B, C, D, G and H), indicating that baculovirus infection did not trigger apoptosis in SL-HP cells under non-starvation condition.

### Infection of baculovirus enhanced starvation-induced autophagy

**Figure 2:**
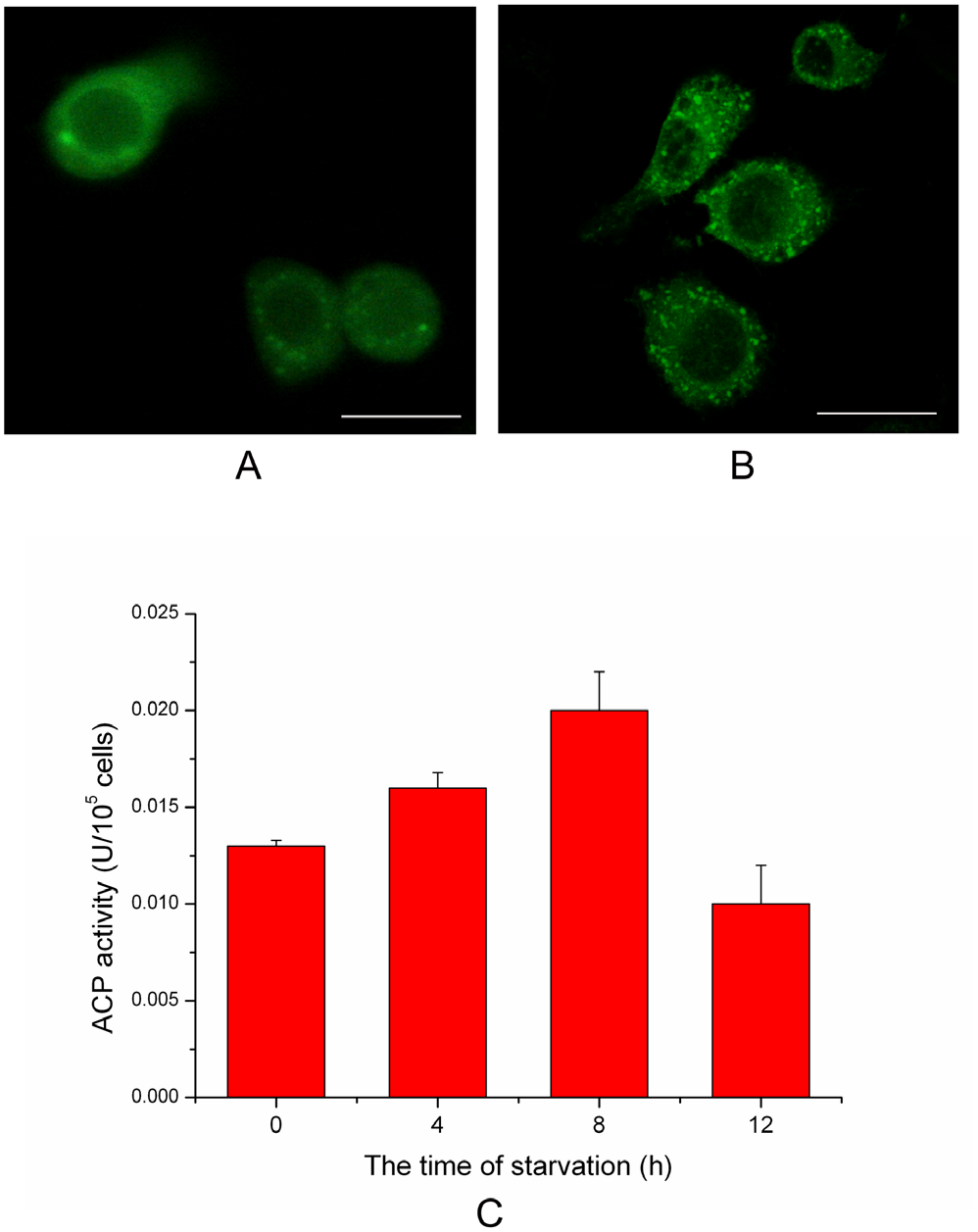
An increase of autophagy activity in starved SL-HP cells. (A) Distribution of GFP-HaAtg8 punctual spots in unstarved SL-HP cells, (B) Distribution of GFP-HaAtg8 punctual spots in SL-HP cells starved for 2 h, showing the increase of autophagosomes in comparison with unstarved SL-HP cells, (C) Acid phosphatase (ACP) activity in SL-HP cells starved for different periods. Bars = 15 µm.

The punctual distribution of GFP-Atg8 and the activity of lyso-enzyme could be used to monitor autophagy [17], [18]. In the present research, GFP-HaAtg8 fluorescence spots increased significantly at 1–2 h of starvation (Figure 2A and B), but decreased at 6 h of starvation (Data not shown). The activity of lyso-enzyme (ACP, acid phosphase) also increased significantly during 0–8 h of starvation, but declined at 12 h of starvation (Figure 2C). Together, these results indicated that autophagy activity increased rapidly in the early stage of starvation (amino acid deprivation).

**Figure 3:**
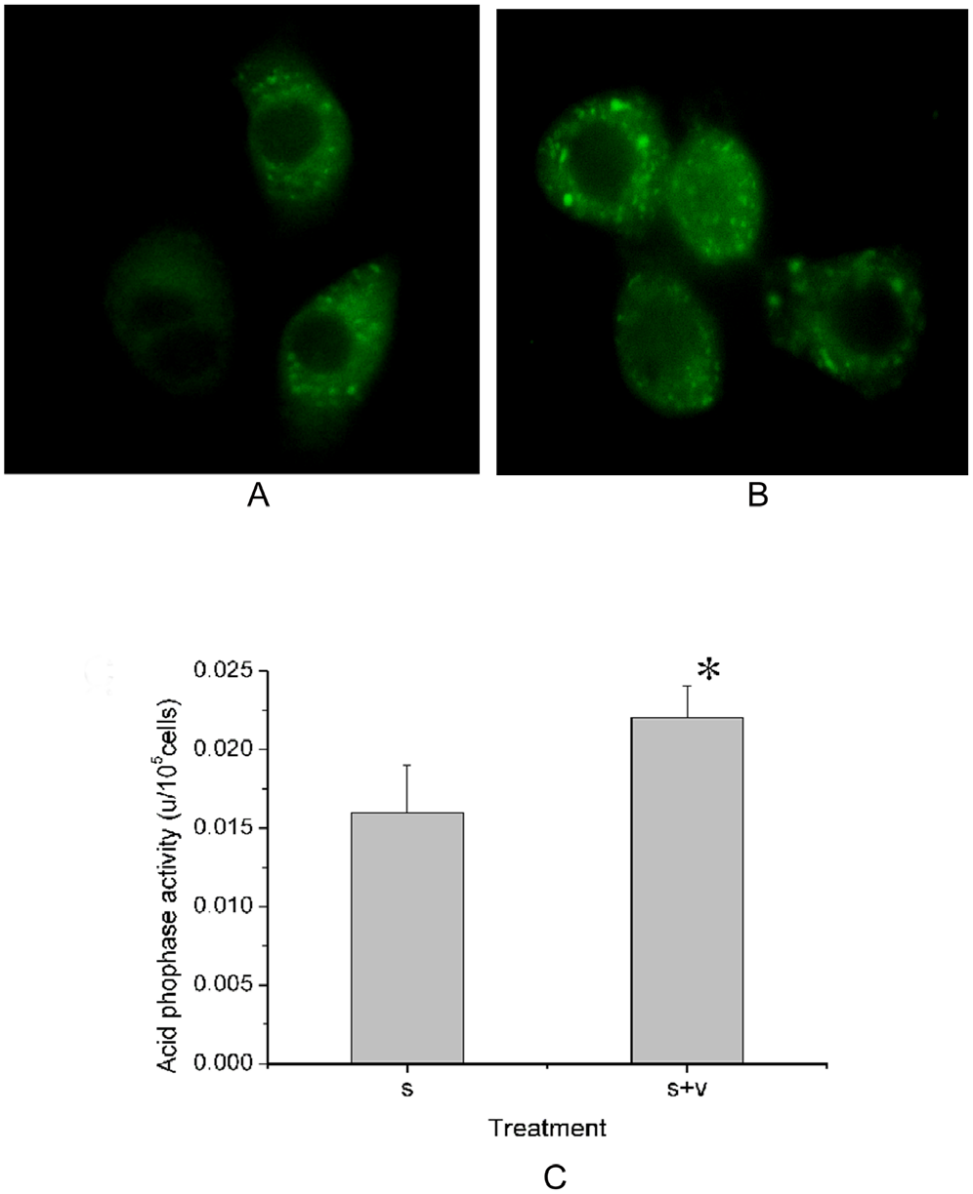
An increase in autophagy activity in AcMNPV-infected SL-HP cells in comparison with non-infected SL-HP cells under starvation pressure. (A) Distribution of GFP-HaAtg8 punctual spots in non-infected SL-HP cells starved for 4.5 h, (B) Distribution of GFP-HaAtg8 punctual spots in AcMNPV-infected SL-HP cells starved for 4.5 h at 2 h of post-infection, showing the increase of autophagosomes in comparison with starved SL-HP cells, (C) Assay of acid phosphatase (ACP) activity between non-infected and infected SL-HP cells starved for 6 h at 4 h of post-infection.

**Figure 4:**
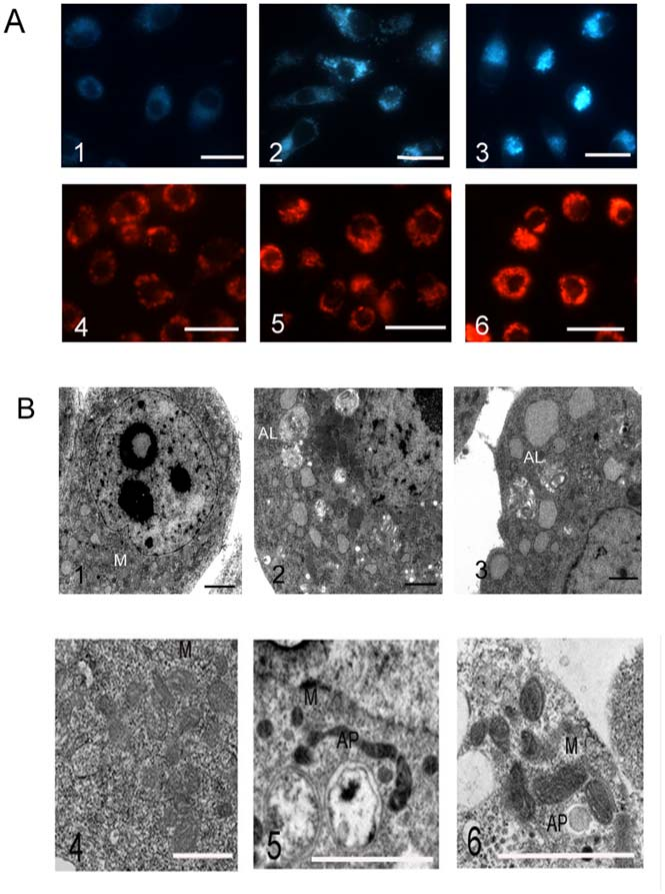
The influences of AcMNPV infection on the starvation-induced autophagy in SL-PHP cells. (A) Staining of MDC (A1–3) and Lyso-tracker Red (A4–6). (A1 and A4) Control SL-HP cells cultured in complete medium with 10% FBS, (A2 and A5) SL-HP cells starved for 6.5 h, (A3 and A6) SL-HP cells infected for 5 h under starvation (6.5 h). (B) Observation of autolysosomes and autophagosomes using electron microscopy. (B1) Control SL-HP cells cultured in complete medium with 10%FBS, (B2) SL-HP cells starved for 6.5 h, showing autolysosomes; (B3) SL-HP cells infected for 5 h under starvation (6.5 h), showing autolysosomes; (B4) Control Sl-zsu-1cells cultured in complete medium with 10% FBS; (B5 and B6) Sl-zsu-1 cells infected with AfMNPV at 4 h of post infection, showing two or one autophagosomes, respectively. M, mitochondria; AL, autolysosomes; AP, autophagosomes. Bars = 20 µm in Fig. A; Bars = 2 µm in Fig. B.

There were more GFP-HaAtg8 fluorescence spots in the AcMNPV-infected SL-HP cells than that in the non-infected cells in the early stage of starvation (Figure 3A and B). The ACP activity also increased significantly when the starved SL-HP cells were infected with AcMNPV at 4 h of post-infection (Figure 3C). The dots with positive MDC staining in cells are correlated with the increase in autophagy activity [9], [18]. In the present study, MDC staining revealed that infection of AcMNPV resulted in the increase of quantity of positive MDC-staining spots in the cells at the early stage of starvation (Figure 4A_1–3_). The staining of lysosomes with Lyso-tracker Red also revealed that the volume and intensity of red fluorescense in the baculovirus-infected cells became larger and stronger under starvation compared with those in the starved cells alone (Figure 4A_4_–_6_). Electron microscopy also revealed that there were more autolysosomes in the starved cells and the infected cells under starvation pressure than those in the cells cultured in complete medium with 10% FBS (Figure 4B_1–3_).

All together, these data demonstrated that infection of baculovirus enhanced a starvation-induced autophagy in the early stage of amino acid starvation.

### SL-HP cells underwent apoptosis following autophagy during baculovirus infection under amino acid deprivation

**Figure 5:**
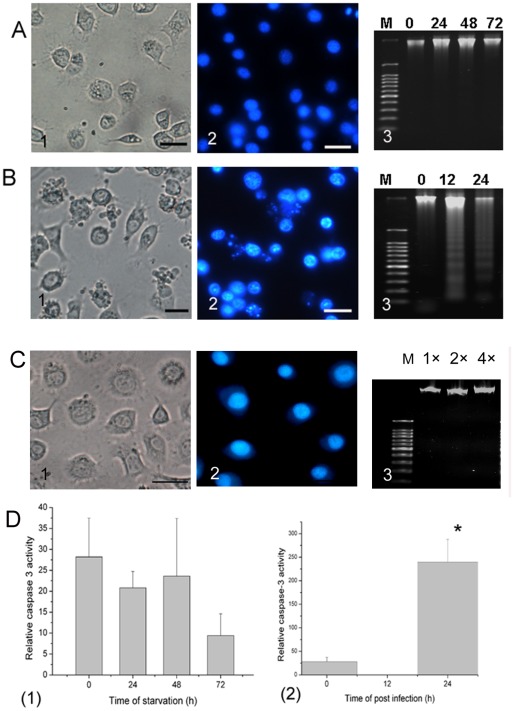
A shift from autophagy to apoptosis triggered by baculovirus under amino acid deprivation. The starved SL-HP cells (A1–3) and the infected cells under starvation (B1–3) were shown. (A1) Observation of the cells starved for 48 h using light microscope, (A2) Hoechst staining of the cells starved for 48 h; (A3) DNA agarose electrophoresis showing that no DNA degradation occurred after 0, 24, 48 and 72 h of starvation. (B1) Observation of the cells infected for 24 h using light microscope, (B2) Hoechst staining of the cells infected for 24 h under starvation (25.5 h), (B3) DNA agrose electrophoresis showing that DNA fragmentation occurred in AcMNPV-infected SL-HP cells under starvation at 12 h or 24 h of post-infection, (C1) unstarved SL-HP cells, (C2) unstarved SL-HP cells stained with Hoechst; (C3) DNA extracted from SL-HP cells starved for 24 h was run at 1×, 2× and 4×; (D) Analysis of relative caspase-3 activity, (D1) Starved cells; (D2) AcMNPV-infected cells under starvation condition. *p<0.05. Bars = 20 µm. M, 100 bp DNA ladder.

To eliminate the possibility that amino acid deprivation causes cell death or apoptosis, trypan blue staining was used to reveal the mortality of cells during the starvation from 0 to 72 h, and no significant difference was seen between the starved cells and normal cells cultured in complete medium with 10% FBS (data not shown). Agarose electrophoresis of DNA revealed that there was no cleavage of nuclear DNA into oligonucleosomal fragments in the starved cells during this period (Figure 5A_1–3_ and 5C_3_). No significant caspase-3 activity was detected in the starved cells during the period of starvation (Figure 5D_1_). These data suggested that the starved cells had not undergone apoptosis under amino acid deprivation for at least 72 h.

The cells starved for 1.5 h were infected with AcMNPV at a moi = 5, and characteristic of apoptosis was analyzed. At 9 h of post-infection, the cells began to show typical hall-markers of apoptosis, including formation of apoptotic bodies (Figure 5B_1_), condensation of chromatin (Figure 5B_2_), cleavage of nucleosomes shown by DNA ladder (Figure 5B_3_), and activation of caspase-3 (Figure 5D_2_)

### Autophagy was also followed by apoptosis in unstarved Sl-zsu-1 cells infected with AcMNPV

Since apoptosis followed the autophagy when SL-HP cells were challenged with baculovirus infection under starvation as described above, we would further confirm whether the infection of baculovirus could promote autophagy activity in normal condition. To test this, AfMNPV was used to infect the Sl-zsu-1 cell line from *S. litura* cultured in complete medium with 10% FBS. The transmission electronic microscope revealed that the autophagosomes were obseved in the early stage of apoptosis induced with baculovirus at 4 h of post-infection (Figure 4B_4_–_6_). These results indicated that baculovirus-induced apoptosis also followed autophagy in non-permissive Sl-zsu-1 cell line under non-starvation condition.

### Baculovirus-induced apoptosis did not result from differential expression of the ie-1 and p35 protein under amino acid deprivation

**Figure 6:**
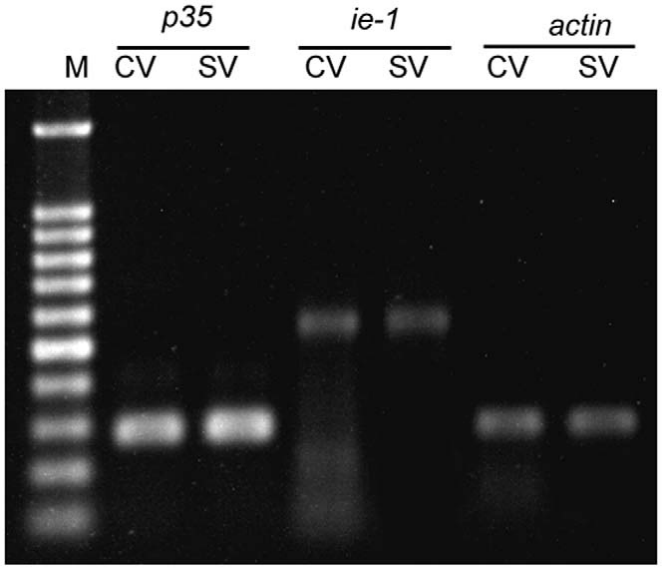
The expression analysis of *p35* and *ie-1* genes from AcMNPV using semi-q-PCR in unstarved and starved SL-HP cells, respectively. CV: cells cultured in complete medium with 10% FBS and infected for 6 h; SV: cells infected for 6 h under starvation (7.5 h), and *actin3A* gene was used as internal control.

The apoptotic gene *ie-1* and the anti-apoptotic gene *p35* could regulate insect cell apoptosis. If the expression of *ie-1* is up-regulated or the expression of *p35* is down-regulated in baculovirus-infected permissive cells, apoptosis will occur. However, the analysis of semi-quantitative PCR results showed that the transcription level of the *ie-1* and the *p35* genes did not change significantly at mRNA level at 6 h of post-infection under starvation pressure in comparison with the expression of the two genes in the unstarved SL-HP cells infected with AcMNPV (Figure 6). Thus, baculovirus-induced apoptosis did not result from differential expression of the ie-1 and p35 protein under amino acid deprivation.

### Mitochondria was involved in apoptosis

**Figure 7:**
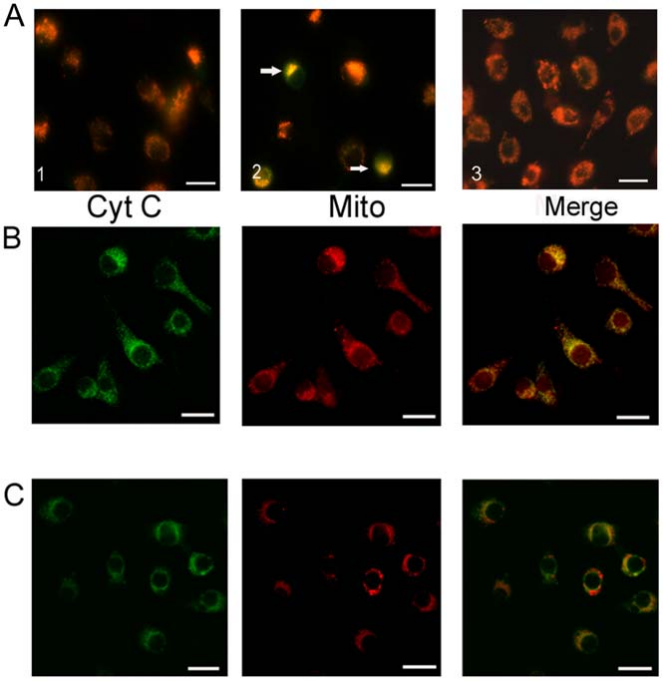
The JC-1 staining of mitochondria (A) and co-localization of mitochondria with cytochrome c (B and C) in apoptosis induced with AcMNPV in SL-HP cells under amino acid deprivation. (A1) SL-HP cells starved for 6.5 h, (A2) SL-HP cells infected for 5 h under starvation (6.5 h), showing damaged mitochondria with yellow fluorescence, (A3) unstarved SL-HP cells, (B) SL-HP cells starved for 6.5 h showing the co-localization of cytochrome *c* with mitochondria, (C) SL-HP cells infected for 5 h under starvation (6.5 h); no co-localization of cytochrome c with mitochondria was observed. Arrow pointed to damaged mitochondria. Bars = 20 µm.

To investigate whether mitochondria are involved in baculovirus-induced apoptosis, JC-1 staining was used to detect the integrity of mitochondria membrane. As shown in the Figure 7A_1_ and A_3_, the red mitochondria were observed in both starved and unstarved cells. However, in the early stage of AcMNPV-induced apoptosis under amino acid deprivation, the yellow fluorescent mitochondria which were merged from green and red fluorescence increased greatly after JC-1 staining, suggesting that the impaired mitochondria accumulated in cells after AcMNPV infection (Figure 7A_2_). Confocal laser microscope also revealed that cytochrome c was released from mitochondria into cytoplasm as shown by the analysis of co-localization of mitochondria with cytochrome c (Figure 7B and C). Thus, the release of cytochrome c was involved in the pathway of apoptosis triggered by baculovirus in our present study.

### No cleavage of SlAtg6 occurred during apoptosis of SL-HP cells

**Figure 8:**
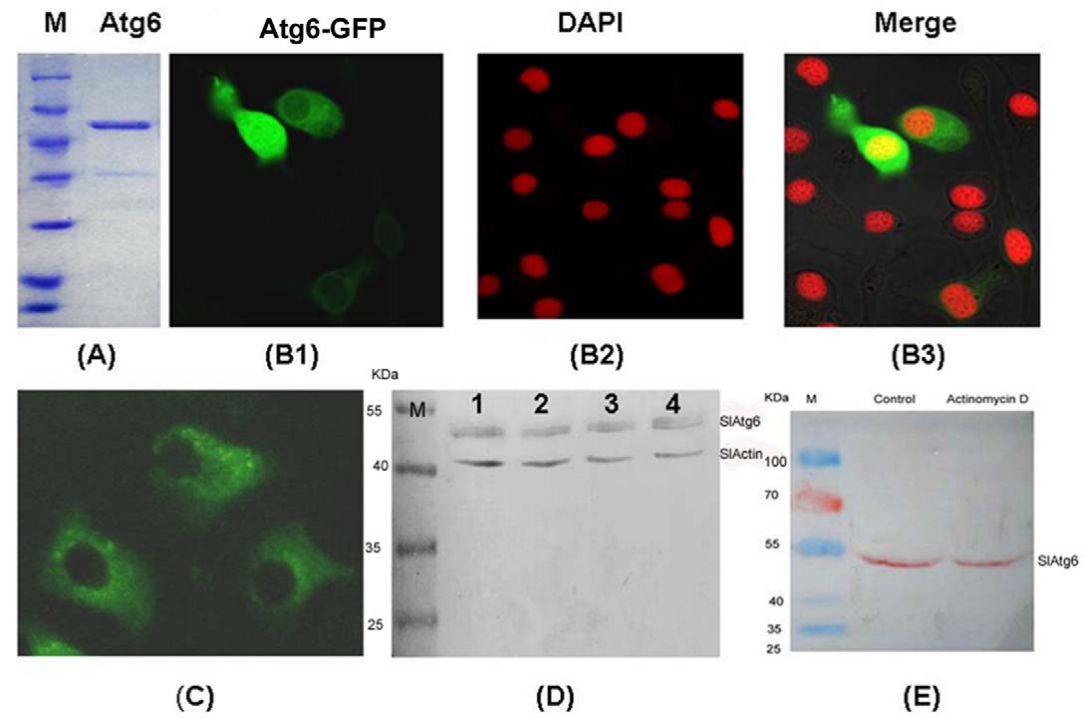
The expression, localization, abundance and cleavage of Atg6 in insect cells. (A) the purified BmAtg6-His/V5 expressed in *E.coli*, (B) LD652 cells transfected with plasmid BmAtg6-GFP and observed with the confocal laser microscopy at 24 h post transfection, showing the localization of over-expressed BmAtg6-GFP protein; The blue light emitted by Hoechst was changed to red light with software, (C) localization of the endogenous SlAtg6 in SL-HP cells shown by immunofluorescence using anti-BmAtg6 mouse serum. (D)Abundance alteration of SlAtg6 under various conditions; lane 1, unstarved Sl-HP cells; lane 2, SL-HP cells starved for 6 h; lane 3, AcMNPV-infected SL-HP cells at 4 h of post-infection; lane 4, AcMNPV-infected SL-HP cells starved for 6 h at 4 h of post-infection, (E) No cleavage of endogenous SlAtg6 was observed in normal SL-HP cells (control) and apoptotic cells induced with actinomycin D (1 µg/ml) by western blot using anti-BmAtg6 serum. Bars = 20 µm.

Since Atg6 is putative protein in lepidopteran at present [24], therefore, it is necessary to know whether Atg6 is expressed and where it located in lepidopteran cells. The BmAtg6 were expressed in *E. coli* and purified via Ni- NTA column in order to prepare anti-BmAtg6 serum (Figure 8A). To visualize Atg6 protein in lepidopteran cells, a *B. mori* BmAtg6-GFP fusion protein under the control of the *OP-IE2* promoter of the OpMNPV was expressed in LD652 cells (the construction was described in the methods). The confocal laser microscope revealed that the over-expressed BmAtg6-GFP protein was mainly located in cytoplasm and to some extent in nucleus in LD652 cells (Figure 8B), which was similar with the localization of mammalian Atg6/Beclin-1. Endogenous *S. litura* Atg6 (SlAtg6) was detected via immunofluorescence using anti-BmAtg6 mouse serum in SL-HP cells, and its localization in SL-HP cells was similar with that of the exogenous BmAtg6-GFP in LD652 cells (Figure 8C). The western-blot assay revealed that the abundance of SlAtg6 protein did not alter in SL-HP cells under various conditions (Figure 8D). SL-HP cells underwent apoptosis when they had been treated with actinomycin D at the final concentration of 1 µg/ml for 12 h (data not shown). Unlike human Beclin-1/Atg6 protein [14], no cleavage of SlAtg6 in SL-HP cells was observed by analysis of western blot when apoptosis was induced with actinomycin D (Figure 8E). The result suggested that dysfunction of mitochondria did not result from the cleavage of SlAtg6.

## Discussion

In our present experiments, AcMNPV infection did not block autophagy in starved SL-HP cells, but enhanced autophagy activity in some degree, as shown by the staining of lysosome tracker, analysis of lyso-enzyme activity, observation of electronic microscope and assay of punctual spots of GFP-HaAtg8 in SL-HP cells in the early stage of starvation. However, the mechanism is not clear. It has been reported that autophagy activity increased in host cells when some bacteria or viruses infected them [25]–[27]. In the present research, the interaction of proteins from baculovirus envelope with cell membrane at the first step of infection, the released proteins from baculovirus nucleocapsid or others might mediate the signal transduction pathway leading to autophagy in the starved SL-HP cells.

Starvation could trigger autophagy *in Vivo* [28], reduce baculovirus replication and delay the death of insect larvae such as *B. mori* and *Heliothis virescens* [29]. In the present study, baculovirus triggered apoptosis in the permissive cells when the cells were starved. The shift from permissive to non-permissive may be one of important mechanisms for the delay of insect larvae death when baculovirus infection followed starvation.

No literature is available about the effects of baculovirus infection on lepidopeteran autophagy. In the present research, Lyso-track Red and MDC staining, the analysis of acid phosphatase activity and the observation of transmission electronic microscopy demonstrated that baculovirus infection enhanced autophagy followed by apoptosis in the early stage of infection.

Our previous studies have demonstrated that the mitochondria play a key role in lepidopteran insect cell apoptosis [11], [30]. In the current study, mitochondria lost membrane potential and cytochrome c was released from mitochondria into cytoplasm in the early stage of apoptosis, suggesting that mitochondria were involved in apoptosis triggered by baculovirus infection under amino acid deprivation.

At present, the pathway of AcMNPV-induced apoptosis in permissive SL-HP cells under starvation is unclear. Many reports showed that apoptosis was regulated via expression of *ie-1* and *p35* and the balance between the ie-1 and p35 protein plays a very important role in the replication of baculovirus [22]. The *ie-1* and *p35* genes also co-operate in the activation of baculovirus AcMNPV and HzNV-1 promoters [31] In addition, *iap1* and *iap2* genes of baculovirus also play a key role in insect cell apoptosis [32]. In the present study, our data showed that the expression level of *ie-1* or *p35* did not change significantly under amino acid deprivation in the early stage of infection (6 h) compared to the AcMNPV-infected cells cultured in complete medium with 10% FBS, indicating that apoptosis induced with AcMNPV might not result from the differential expression of *ie-1* or *p35* genes under normal or starvation condition at the early stage of infection (6 h).

**Figure 9:**
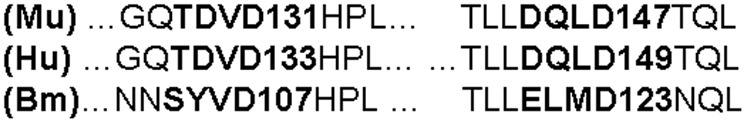
Comparison of possible cleavage sites of caspases between mammalian Atg6/Beclin 1 and lepidopteran Atg6. Mu: mouse Atg6; Hu: human Atg6; Bm: *Bombyx mori* Atg6.

On the other hand, many autophagy-specific proteins bridge autophagy and apoptosis. Cleavage of a few autophagy-specific proteins such as Beclin-1/Atg6 activated the signal pathway of cell apoptosis mediated by mitochondria in mammalian cells [14], [33]–[35]. In our present experiments, the SlAtg6 protein was detected in SL-HP cells by western blot using anti-BmAtg6 mouse serum, suggesting that SlAtg6 possesses high homology with BmAtg6 (Figure 8). No cleavage of SlAtg6 was observed in this western blot analysis. The comparison of protein sequences between BmAtg6 and mammalian Atg6/Beclin-1 revealed that the sequences of cleavage site of mammalian Atg6 differed from those of BmAtg6 (Figure 9). Caspases recognize and cleave Beclin-1 but not SlAtg6, suggesting that the role of SlAtg6 in cross-talking between autophagy and apoptosis might be different from that in mammalian.

AcMNPV baculovirus infection can not induce apoptosis in unstarved SL-HP cells with baseline autophagy activity. However, when autophagy activity increased under amino acid deprivation in the early stage of starvation, AcMNPV infection induced apoptosis in the starved SL-HP cells with mitochondrial dysfunction. It suggests that the increased autophagy triggered by starvation enhances apoptosis induced by AcMNPV, and mitochondrial dysfunction is involved in apoptosis. However, the mitochondrial dysfunction is not mediated either by the cleavage of SlAtg6 or the differential expression of *ie-1* and *p35* between the unstarved and starved SL-HP cells.
